# Structural Profiling of Bacterial Effectors Reveals Enrichment of Host-Interacting Domains and Motifs

**DOI:** 10.3389/fmolb.2021.626600

**Published:** 2021-05-03

**Authors:** Yangchun Frank Chen, Yu Xia

**Affiliations:** Department of Bioengineering, McGill University, Montreal, QC, Canada

**Keywords:** structural biology, bacterial effector, host-pathogen interaction, protein-protein interaction, globular domains, short linear motifs, structural homology, convergent evolution

## Abstract

Effector proteins are bacterial virulence factors secreted directly into host cells and, through extensive interactions with host proteins, rewire host signaling pathways to the advantage of the pathogen. Despite the crucial role of globular domains as mediators of protein-protein interactions (PPIs), previous structural studies of bacterial effectors are primarily focused on individual domains, rather than domain-mediated PPIs, which limits their ability to uncover systems-level molecular recognition principles governing host-bacteria interactions. Here, we took an interaction-centric approach and systematically examined the potential of structural components within bacterial proteins to engage in or target eukaryote-specific domain-domain interactions (DDIs). Our results indicate that: 1) effectors are about six times as likely as non-effectors to contain host-like domains that mediate DDIs exclusively in eukaryotes; 2) the average domain in effectors is about seven times as likely as that in non-effectors to co-occur with DDI partners in eukaryotes rather than in bacteria; and 3) effectors are about nine times as likely as non-effectors to contain bacteria-exclusive domains that target host domains mediating DDIs exclusively in eukaryotes. Moreover, in the absence of host-like domains or among pathogen proteins without domain assignment, effectors harbor a higher variety and density of short linear motifs targeting host domains that mediate DDIs exclusively in eukaryotes. Our study lends novel quantitative insight into the structural basis of effector-induced perturbation of host-endogenous PPIs and may aid in the design of selective inhibitors of host-pathogen interactions.

## Introduction

An important goal of systems microbiology is to understand how host-pathogen protein-protein interactions (PPIs) impact host-endogenous signaling networks. Effector proteins are virulence factors secreted by pathogenic bacteria and injected directly into the host cytoplasm via specialized secretion systems (Galan, 2009). Effectors are key mediators of host-pathogen interactions throughout the infection cycle, from initial host attachment and pathogen internalization, to migration and proliferation in the host. Among the diverse biochemical activities of effectors discovered so far are guanine nucleotide exchange factors and dissociation inhibitors, GTPase-activating proteins, kinases and phosphatases, ubiquitin ligases, and so on (Fu and Galan, 1998; Steele-Mortimer et al., 2000; Janjusevic et al., 2006). A common virulence mechanism of effectors is functional mimicry of host activities, whereby effectors compete with host proteins for control of host signaling pathways. This functional mimicry can be achieved in one of two ways: horizontal acquisition of eukaryotic globular domains, or convergent evolution of domains and short linear motifs in bacteria that bear little sequence or structural similarity to eukaryotic proteins (Stebbins and Galan, 2001; Popa et al., 2016; Scott and Hartland, 2017). These structural modules allow effectors to interact seamlessly with host-endogenous factors involved in actin remodelling, protein degradation and cell cycle regulation, helping the pathogen to survive and thrive in the host while bypassing immune surveillance. Previous studies have uncovered a large repertoire of bacterial effectors that are structural homologs of eukaryotic proteins, giving rise to models for predicting effectors based on the premise that whereas most domains are uniformly distributed among all species of bacteria, eukaryotic-like domains are overrepresented in the genomes of pathogenic and symbiotic species (Jehl et al., 2011; Marchesini et al., 2011). Although useful for identifying candidate effectors in metagenomic analyses, a main caveat of these models is their treatment of domains as individual, rather than interacting, entities that contribute to protein-protein interactions. Eukaryotic domains and their domain-domain interaction (DDI) partners are of special interest to the study of host-pathogen interactions, as they are often mimicked or targeted by pathogens to subvert host signaling pathways (Arnold et al., 2012). Despite studies pointing to the presence of many eukaryotic-like domains in bacterial effectors, there has yet to be a comprehensive, quantitative analysis of the relevance of such domains to host-endogenous PPIs, which is crucial to understanding systems-level changes in the host upon infection with pathogens.

Past studies on host-bacteria protein-protein interactions (PPIs) have examined either individual interactions at the domain level (Cazalet et al., 2004), or interactome networks at the whole protein level (Schweppe et al., 2015), but never both at the domain level and on an interactome scale. In this work, we ask the following new question: how do host-bacteria PPIs mimic and modulate host-endogenous PPIs at the protein domain level on an interactome scale? To answer this question, we carried out two analyses of host-interacting bacterial proteins: the first on mimicry of host-endogenous binding sites by bacterial effectors, and the second on enrichment of host-interacting domains and short linear motifs in bacterial effectors. In the first analysis, we examined the mechanism of host binding site mimicry by bacterial proteins where, rather than creating new binding sites, bacteria recruit existing binding sites involved in host-endogenous PPIs for host-bacteria PPIs (Cazalet et al., 2004). Previous studies on host-virus interactions found that while two human proteins sharing binding sites on a common target tend to be structurally similar, a viral protein and a human protein sharing binding sites on a common target tend to be structurally distinct (Franzosa and Xia, 2011; Garamszegi et al., 2013). In other words, binding site sharing among human proteins is largely attributable to divergent evolution through gene duplication, whereas binding site mimicry by viral proteins tends to involve convergent evolution of unique host-interacting modules in viruses. To our knowledge, similar analyses have yet to be performed for host-bacteria interactions. In the second analysis, we tested the hypothesis that compared to non-effector proteins, bacterial effectors are enriched for domains that either mimic or target host domains involved in eukaryote-specific domain-domain interactions (DDIs). In addition to domains, we also tested whether effectors tend to contain a higher variety and density of short linear motifs that interact with host domains mediating DDIs exclusively in eukaryotes.

## Results

### Mechanism of Binding Site Sharing in Host-Endogenous *vs.* Host-Bacteria Protein-Protein Interaction Network

Previous studies have established binding site mimicry via convergent evolution as a key feature of human-virus PPIs where, rather than securing new binding sites, viruses have evolved unique, non-host-like structural domains and short linear motifs to compete with host proteins for the same binding sites on a common host target (Franzosa and Xia, 2011; Garamszegi et al., 2013). As bacteria and viruses are both known to hijack host molecular machinery through interacting with host proteins, we performed similar analyses on a domain-resolved host-bacteria PPI network with regard to binding site mimicry and its evolutionary mechanism. To this end, we acquired eukaryote-endogenous (within animals/plants/fungi), bacteria-endogenous and host-bacteria (between animals/plants and pathogenic bacteria) PPI data, and resolved each PPI into domain-domain interactions (DDIs) between interacting proteins, based on DDI templates previously derived from 3D structures of protein complexes (Materials and Methods). The resulting domain-resolved, eukaryote-bacteria PPI network consists of: 1) 57,019 PPIs among 22,110 eukaryotic proteins, resolved into 4,953 DDIs among 2,859 eukaryotic domains; 2) 3,362 PPIs among 3,000 bacterial proteins, resolved into 1,434 DDIs among 1,120 bacterial domains; and 3) 173 PPIs between 107 host proteins and 103 bacterial proteins, resolved into 87 DDIs between 53 host domains and 63 bacterial domains. The entire list of domain-resolved host-bacteria, bacteria-endogenous, and eukaryote-endogenous PPIs can be found in Supplementary File S1.

**FIGURE 1:**
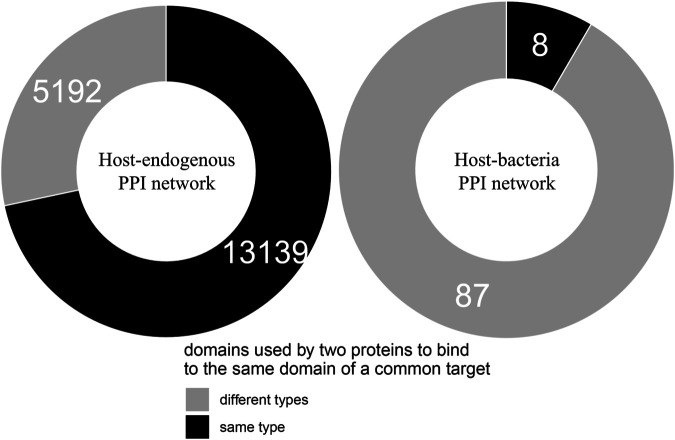
Different evolutionary mechanisms for binding site sharing in the host-endogenous vs. host-bacteria PPI network. Two host proteins are more likely to use the same type of domain to bind to the same domain of a common target, suggesting divergent evolution followed by gene duplication. A host protein and a bacterial protein are more likely to use different types of domain to bind to the same domain of a common host target, suggesting convergent evolution (or extreme divergent evolution) of non-host-like domains in bacteria. The difference in dominant evolutionary mechanisms is statistically significant (Fisher’s exact test, two-tailed *p* < 2.2 * 10^−16^).

We found that of the 103 host-targeting bacterial proteins, 95 (92%) bind to the same domains on their host target that are otherwise bound by host-endogenous proteins, suggesting that like viruses, bacteria also tend to recruit domains involved in host-endogenous PPIs for host-pathogen PPIs (Cazalet et al., 2004). We then determined whether bacterial and host proteins binding to the same domain on another host protein are structurally similar. We found that of 18,331 cases where two host proteins A and B bind to the same domain on a common target, 13,139 (72%) cases involve domains which are conserved between A and B, while in the remaining 5,192 (28%) cases there is no domain conserved between A and B. Conversely, among 95 cases where host protein X and bacterial protein Y bind to the same domain on another host protein, only 8 cases (8%) involve domains which are conserved between X and Y, while in the remaining 87 (92%) cases there is no domain conserved between X and Y. In other words, compared to binding site sharing among host proteins, binding site mimicry by bacterial proteins appears significantly more likely to involve convergent evolution (or extreme divergent evolution) of bacteria-exclusive domains, rather than horizontal acquisition of host domains (Fisher’s exact test, two-tailed *p* < 2.2 * 10^−16^). Figure 1 shows the contrast in dominant evolutionary mechanisms behind binding site sharing in the host-endogenous *vs.* host-bacteria PPI network.

### Effectors Structurally Mimic Host Domains Involved in Eukaryote-Specific Protein-Protein Interactions

Having examined the mechanism of binding site mimicry in the host-bacteria PPI network, we then asked whether bacterial effectors tend to mimic or target host domains that mediate DDIs predominantly in eukaryotes, as opposed to domains that mediate DDIs in eukaryotes and bacteria with similar likelihood ‐ the rationale being that the former are involved in eukaryote-specific processes such as protein ubiquitination (Grau-Bove et al., 2015), which are prime targets for pathogens to manipulate, whereas the latter are involved in highly conserved, essential cellular processes (Walhout et al., 2000; Matthews et al., 2001), which are unlikely to be perturbed in host-pathogen interactions. We found that of the 63 host-binding bacterial domains in our dataset, 12 have homologs in eukaryotes, among which 7 mediate DDIs exclusively in eukaryotes (PF12796, PF00092, PF12799, PF02205, PF04564, PF00646, PF13676), 3 mediate DDIs primarily in eukaryotes (PF00069, PF00583, PF00183), and 2 have similar numbers of DDI partners in eukaryotes and bacteria (PF13472, PF00085) (Supplementary File S2). Meanwhile, of the 31 host domains targeted by bacteria-exclusive domains, 28 otherwise mediate DDIs exclusively in eukaryotes, 2 mediate DDIs primarily in eukaryotes, and 1 has similar numbers of DDI partners in eukaryotes and bacteria (Supplementary File S3). In summary, effectors tend to mimic or target host domains that mediate DDIs predominantly in eukaryotes. Given that effectors comprise nearly half (43/103) of the host-targeting bacterial proteins in our PPI dataset, we hypothesized that compared to the rest of the pathogen proteome, effectors are generally enriched for: 1) eukaryotic-like domains that mediate DDIs predominantly in eukaryotes; and 2) bacteria-exclusive domains that target host domains which, when not involved in host-pathogen DDIs, mediate DDIs exclusively in eukaryotes. To test this hypothesis, we systematically compared 238 effectors and 3,921 non-effectors with unique domain signatures, encoded by 84 bacterial species of verified pathogenicity (Urban et al., 2020). Effectors encoded by the 84 pathogenic species are selected from PHI-base, if a pathogen gene’s “Gene Function” or “Mutant Phenotype” column contains the keyword “effector”, as well as from the UniProt database if the gene name or cellular location contains keywords such as “type * effector”, “t*ss effector”, or “secreted effector”. To ensure the same selection criteria are applied to all proteins, non-effectors encoded by the 84 pathogenic species include proteins not already in the effector set, whose cellular location is any one of cytoplasmic, membrane or secreted (Materials and Methods).

**FIGURE 2:**
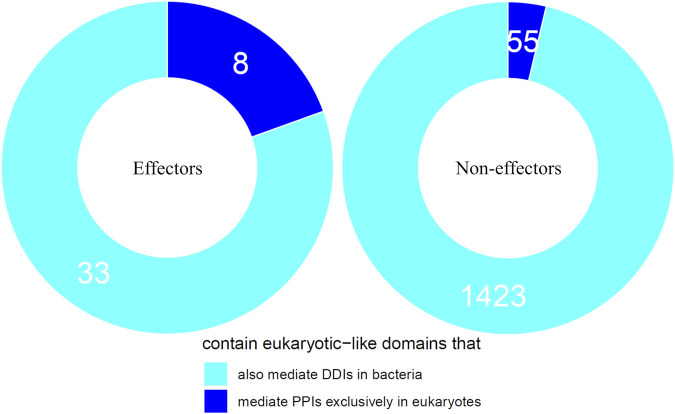
Effectors are enriched for eukaryotic-like domains that mediate PPIs exclusively in eukaryotes. Among pathogen proteins containing domains that mediate experimentally verified PPIs in eukaryotes, 20% effectors and 4% non-effectors contain domains that mediate PPIs exclusively in eukaryotes, suggesting that effectors are six times as likely as non-effectors to repurpose eukaryote-specific processes via eukaryotic-like domains (Fisher’s exact test, two-tailed *p* = 2 * 10^−4^).

**FIGURE 3:**
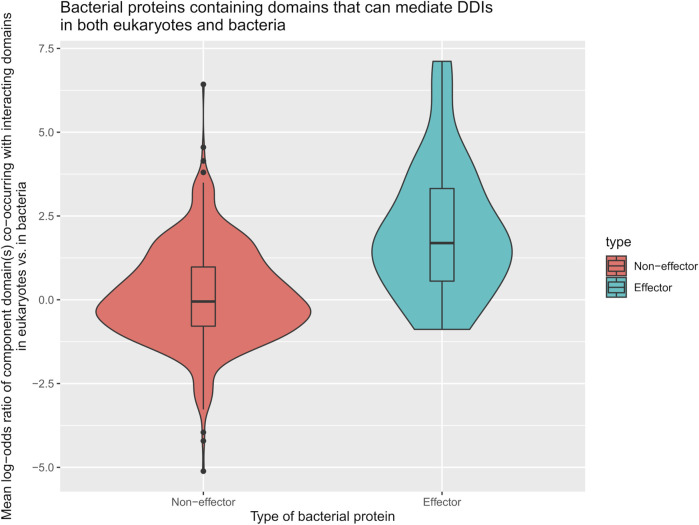
Effectors are enriched for eukaryotic-like domains that mediate PPIs primarily in eukaryotes. Among pathogen proteins containing domains that have DDI partners in both eukaryotes and bacteria, the average domain in effectors is seven times as likely as that in non-effectors to co-occur with DDI partners in eukaryotes rather than in bacteria (Wilcoxon test, two-tailed *p* = 4 * 10^−7^).

We first tested the hypothesis that effectors are enriched for domains that mediate DDIs exclusively in eukaryotes. We found that among 41 effectors and 1,478 non-effectors containing domains that mediate experimentally verified PPIs in eukaryotes, 8 effectors (20%) and 55 non-effectors (4%) contain domains that mediate PPIs exclusively in eukaryotes, suggesting that effectors are six times as likely as non-effectors to repurpose eukaryote-specific processes via eukaryotic-like domains (Fisher’s exact test, two-tailed *p* = 2 * 10^−4^) (Figure 2). Table 1 is a list of effectors containing domains involved in interprotein DDIs in eukaryotes, but neither interprotein nor intraprotein DDIs in bacteria. Next, we tested the hypothesis that effectors are enriched for domains that mediate DDIs primarily in eukaryotes. For domains having DDI partners in both eukaryotes and bacteria, we estimated their propensity for mediating eukaryote-specific DDIs by computing the odds ratio of the domain’s co-occurrence with DDI partners in eukaryotes *vs.* in bacteria. If a bacterial protein contains multiple such domains, we computed a weighted average odds ratio (Table 2). We found that among 26 effectors and 635 non-effectors containing domains that have DDI partners in both eukaryotes and bacteria, the average domain in effectors is seven times as likely as that in non-effectors to co-occur with DDI partners in eukaryotes rather than in bacteria (Wilcoxon test, two-tailed *p* = 4 * 10^−7^) (Figure 3). Table 3 lists effectors with the top 10 highest odds ratios of their component domains co-occurring with DDI partners in eukaryotes rather than in bacteria.

**TABLE 1:** Effectors containing domains that mediate PPIs exclusively in eukaryotes.

UniProt accession	Representative species	Domain(s)	# Pathogenic spp. encoding proteins with domain(s)
B0RMF9	*Xanthomonas campestris*	**PF00560**	14
A0A0A8VF40	*Yersinia ruckeri*	**PF00560**; PF13855	12
A0A199P7E1	*Xanthomonas translucens*	**PF00646**	11
A0A0S4VGA6	*Ralstonia solanacearum*	**PF00646**; PF13516	4
F6G106	*Ralstonia solanacearum*	**PF00646**; PF13516; PF13855	4
A0A1Y0FB05	*Ralstonia solanacearum*	**PF00665**; PF13276	74
A0A286NT26	*Vibrio parahaemolyticus*	**PF02205**	3
D8NFZ7	*Ralstonia solanacearum*	PF01535; PF12854; **PF13812**	1

Domains mediating PPIs exclusively in eukaryotes are marked in bold.

**TABLE 2:** Weighted average host-interacting potential of a multi-domain bacterial protein.

DDI	Eukaryotic species encoding both interacting domains	Eukaryotic species encoding either one or both interacting domains	Bacterial species encoding both interacting domains	Bacterial species encoding either one or both interacting domains	Odds ratio of domain mediating DDIs in eukaryotes *vs.* in bacteria	Weight
A_B	*m*	$H_{1}$	*n*	$B_{1}$	$OR_{1}=\frac{m*\left(B_{1}-n\right)}{n*\left(H_{1}-m\right)}$	$w_{1}=\frac{m*\left(B_{1}-n\right)}{H_{1}+B_{1}}$
A_C	*p*	$H_{2}$	*q*	$B_{2}$	$OR_{2}=\frac{p*\left(B_{2}-q\right)}{q*\left(H_{2}-p\right)}$	$w_{2}=\frac{p*\left(B_{2}-q\right)}{H_{2}+B_{2}}$
D_E	*x*	$H_{3}$	*y*	$B_{3}$	$OR_{3}=\frac{x*\left(B_{3}-y\right)}{y*\left(H_{3}-x\right)}$	$w_{3}=\frac{x*\left(B_{3}-y\right)}{H_{3}+B_{3}}$
Host-interacting potential = $\;\log\left(\frac{\sum_{i=1}^{3}OR_{i}*w_{i}}{\sum_{i=1}^{3}w_{i}}\right)$						

The host-interacting potential of a bacterial protein containing domains A and D, where A and D have DDI partners (domains B, C, E) in both eukaryotes and bacteria, is computed as the Mantel-Haenszel weighted average log odds ratio of domains A and D co-occurring with interacting domains in eukaryotes *vs.* in bacteria. The odds of domain co-occurring with DDI partners are the number of species encoding both interacting domains (*i.e.* DDI is possible) divided by the number of species encoding either one, but not both, of the interacting domains (*i.e.* DDI is not possible).

**TABLE 3:** Effectors containing domains that mediate PPIs primarily in eukaryotes.

UniProt accession	Species	Domain(s)	# Pathogenic spp. encoding proteins with domain(s)	Log odds ratio of domains co-occurring with DDI partners in eukaryotes *vs.* in bacteria
Q5ZRQ0	*Legionella pneumophila*	**PF04564**	1	7.1
O84875	*Chlamydia trachomatis*	**PF02902**	13	6.2
P74873	*Salmonella enterica*	**PF00102**; PF03545; PF09119	2	4.4
Q9KS43	*Vibrio cholerae*	**PF01764**	40	3.9
Q3BQY9	*Xanthomonas euvesicatoria*	**PF13202**; **PF13499**	19	3.8
D8P6Z5	*Ralstonia solanacearum*	**PF13516**	14	3.7
Q8XT98	*Ralstonia solanacearum*	**PF00069**	69	3.5
D2TI55	*Citrobacter rodentium*	**PF00557**	84	2.8
Q8XZN9	*Ralstonia solanacearum*	**PF13516**; **PF13855**	13	2.2
A0A6C9X110	*Escherichia coli*	PF00805; **PF01391**; PF13599	27	2

Domains with DDI partners in both eukaryotes and bacteria are marked in bold.

### Effectors Convergently Target Host Domains Involved in Eukaryote-Specific Protein-Protein Interactions

**FIGURE 4:**
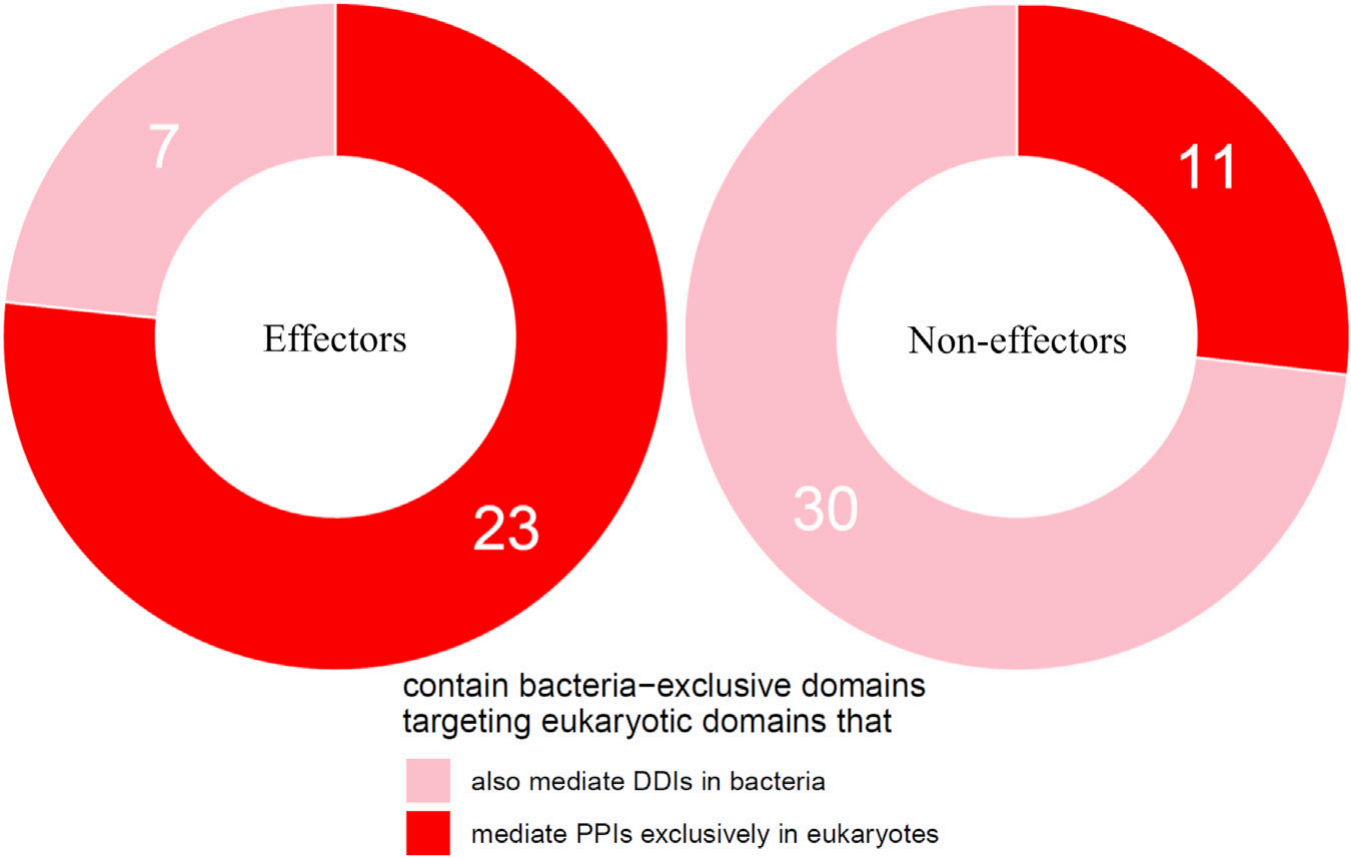
Effectors are enriched for bacteria-exclusive domains targeting host domains that otherwise mediate DDIs exclusively in eukaryotes. Among pathogen proteins containing bacteria-exclusive domains with the potential to target host domains that mediate DDIs in eukaryotes, 77% effectors and 27% non-effectors target host domains that mediate DDIs exclusively in eukaryotes, suggesting that effectors are nine times as likely as non-effectors to disrupt eukaryote-specific processes via bacteria-exclusive domains (Fisher’s exact test, two-tailed *p* = 4 * 10^−5^).

We then tested the hypothesis that effectors are enriched for bacteria-exclusive domains that target host domains which, when not involved in host-pathogen DDIs, mediate DDIs exclusively in eukaryotes. Given that experimental PPI data often suffer from limitations such as false negatives and investigator bias in pathogen selection, we supplemented host-interacting bacteria-exclusive domains supported by PPI data with host-interacting bacteria-exclusive domains supported by interprotein DDI templates (Mosca et al., 2014). In this manner, we identified a total of 207 bacteria-exclusive domains with the potential to target host domains that mediate DDIs in eukaryotes, 52 of which target host domains that mediate DDIs exclusively in eukaryotes. We found that among 30 effectors and 41 non-effectors containing bacteria-exclusive domains with the potential to target host domains that mediate DDIs in eukaryotes, 23 effectors (77%) and 11 non-effectors (27%) target host domains that mediate DDIs exclusively in eukaryotes, suggesting that effectors are nine times as likely as non-effectors to disrupt eukaryote-specific processes via bacteria-exclusive domains (Fisher’s exact test, two-tailed *p* = 4 * 10^−5^) (Figure 4). Supplementary File S4 is a list of effectors with bacteria-exclusive domains targeting host domains that otherwise mediate DDIs exclusively in eukaryotes.

**FIGURE 5:**
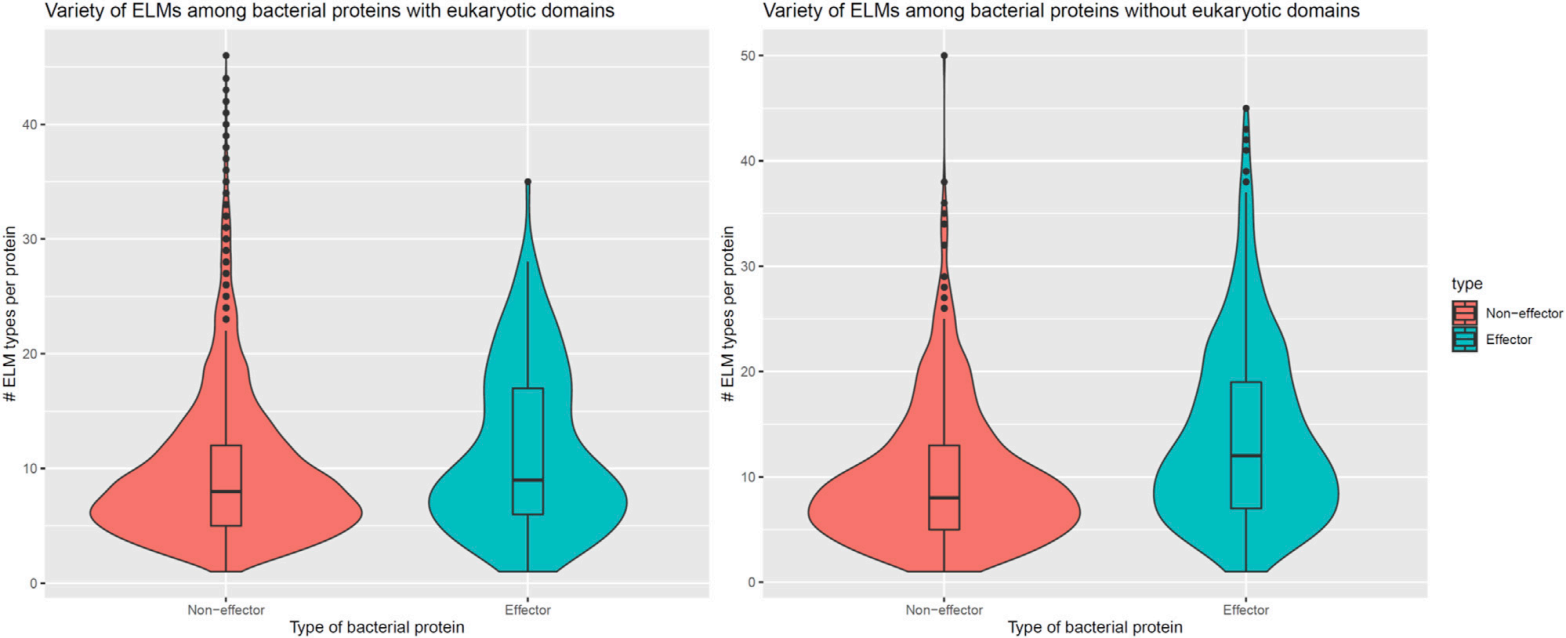
In the absence of eukaryotic-like domains or domains in general, effectors contain a higher variety of eukaryotic linear motifs compared to non-effectors. **Left:** In the presence of eukaryotic-like domains, effectors encode 9 ELM classes per protein, a slightly higher variety (Wilcoxon test, two-tailed *p* = 3 * 10^−3^) compared to 8 ELM classes per non-effector protein. **Right:** In the absence of eukaryotic-like domains or among proteins without Pfam domains, effectors encode 12 ELM classes per protein, a much higher variety (Wilcoxon test, two-tailed *p* < 2.2 * 10^−16^) compared to 8 ELM classes per non-effector protein. ELM = eukaryotic linear motif.

**FIGURE 6:**
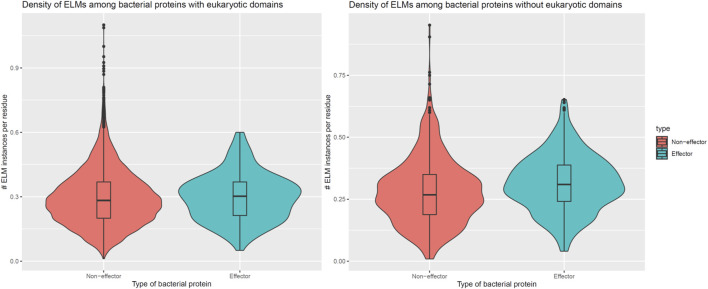
In the absence of eukaryotic-like domains or domains in general, effectors contain a higher density of eukaryotic linear motifs compared to non-effectors. **Left:** In the presence of eukaryotic-like domains, effectors encode 0.30 ELM instances per disordered residue, a slightly higher density that is not statistically significant (Wilcoxon test, two-tailed *p* = 0.3) compared to 0.28 ELM instances per disordered residue in non-effectors. **Right:** In the absence of eukaryotic-like domains or among proteins without Pfam domains, effectors encode 0.31 ELM instances per disordered residue, a higher density that is statistically significant (Wilcoxon test, two-tailed *p* = 2 * 10^−8^) compared to 0.27 ELM instances per disordered residue in non-effectors. ELM = eukaryotic linear motif.

In addition to encoding globular domains that either mimic or target host domains, effectors also encode short linear motifs that bind to host domains with similar specificities as host-endogenous proteins, albeit sharing little homology with the latter (Samano-Sanchez and Gibson, 2020). These short linear motifs follow particular sequence patterns and are predominantly located in intrinsically disordered regions of proteins that are accessible to interacting partners (Davey et al., 2012). To determine whether effectors are enriched for host-interacting motifs, we counted the number of unique classes and instances of eukaryotic linear motifs (ELMs) (Kumar et al., 2020) in long disordered regions of bacterial proteins as annotated by the MobiDB database (Piovesan et al., 2018). When comparing 162 effectors and 8,414 non-effectors with unique ELM compositions and containing eukaryotic-like domains, we found that effectors and non-effectors encode 9 and 8 ELM classes per protein (Figure 5, Left), along with 0.30 and 0.28 ELM instances per disordered residue (Figure 6, Left), respectively. In other words, in the presence of eukaryotic-like domains, effectors encode a slightly higher variety (Wilcoxon test, two-tailed *p* = 3 * 10^−3^), but similar density of ELMs (Wilcoxon test, two-tailed *p* = 0.3) compared to non-effectors. When comparing 521 effectors and 794 non-effectors with unique ELM compositions and not containing eukaryotic-like domains or any Pfam domains, however, we found that effectors and non-effectors encode 12 and 8 ELM classes per protein (Figure 5, Right), along with 0.31 and 0.27 ELM instances per disordered residue (Figure 6, Right), respectively. In other words, in the absence of eukaryotic-like domains or among pathogen proteins without Pfam domains, effectors encode a higher variety (Wilcoxon test, two-tailed *p* < 2.2 * 10^−16^) as well as higher density of ELMs (Wilcoxon test, two-tailed *p* = 2 * 10^−8^) compared to non-effectors.

## Discussion

Pathogenic bacteria have evolved a plethora of strategies to survive and thrive in eukaryotic hosts. A key strategy is functional mimicry of host activities, which is achieved through one of two orthogonal evolutionary mechanisms: horizontal acquisition of eukaryotic domains or convergent evolution of bacteria-exclusive domains (Stebbins and Galan, 2001; Popa et al., 2016; Scott and Hartland, 2017). Current literature contains many case studies of bacterial effectors targeting host domains involved in host-endogenous PPIs via eukaryotic-like domains or bacteria-exclusive domains. For instance, *Ralstonia solanacearum* have acquired a host-like F-box domain (PF00646) that competes with host-endogenous F-box protein for binding to SKP1, thus hijacking the ubiquitin-proteasome pathway in *Arabidopsis thaliana* (Angot et al., 2006), while *Shigella flexneri* have convergently evolved a GEF domain (PF03278) that competes with host Rho GEF, thus activating the Rho GTPase signaling pathway in humans (Huang et al., 2009). In addition to mechanistic studies on individual host-targeting domains in bacteria, databases of eukaryotic-like domains and short linear motifs provide a snapshot of the extent to which bacterial pathogens mimic host structural modules. For instance, EffectiveDB, a database for predicting bacterial effectors based on several criteria including the presence of eukaryotic-like domains, currently reports 2,636 eukaryotic-like domains as being significantly enriched (*Z*-score ≥ 4) in the genomes of pathogenic *vs.* non-pathogenic bacteria (Eichinger et al., 2016). Meanwhile, the Eukaryotic Linear Motif Resource currently contains ∼100 instances of bacteria-mimicked eukaryotic short linear motifs from a small number of extensively studied pathogenic species (Samano-Sanchez and Gibson, 2020).

**FIGURE 7:**
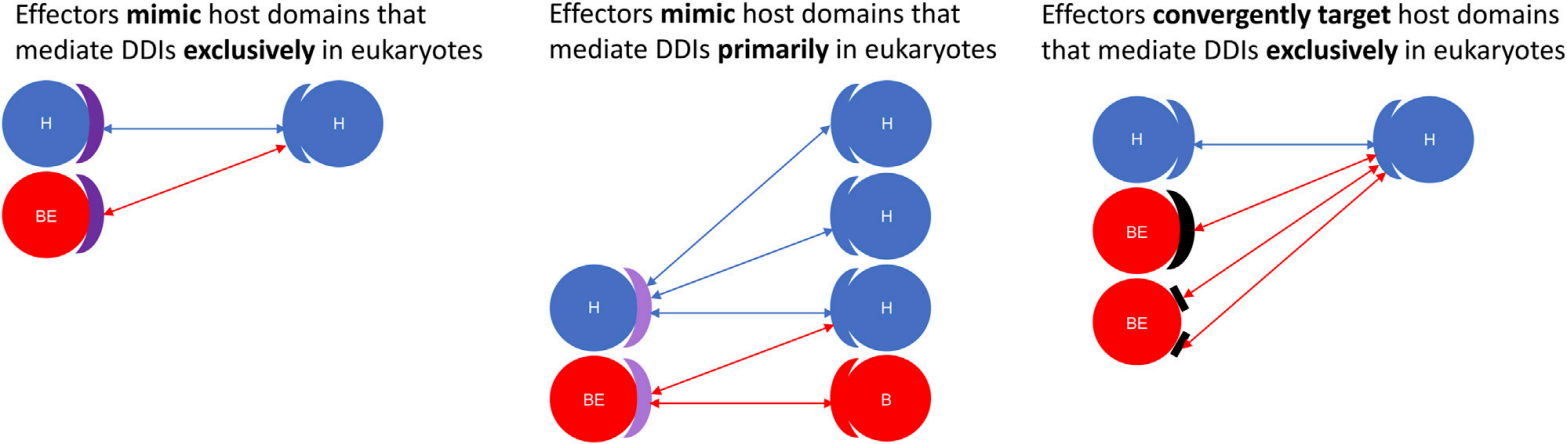
Effectors are enriched for structural modules that either mimic or target host domains mediating eukaryote-specific domain-domain interactions. Using a domain-resolved eukaryote-bacteria protein-protein interaction network, we identified domains that mediate PPIs exclusively in eukaryotes (Left: deep purple arc), as well as domains that are more likely to co-occur with domain-domain interaction partners in eukaryotes rather than in bacteria (Middle: light purple arc). We found that compared to generic proteins encoded by pathogenic bacteria, effector proteins are significantly enriched for domains and short linear motifs that either mimic or target host domains mediating DDIs either exclusively or primarily in eukaryotes. **Left:** Effectors mimic host domains that mediate DDIs exclusively in eukaryotes; **Middle:** Effectors mimic host domains that mediate DDIs primarily in eukaryotes; **Right:** Effectors convergently evolve domains and short linear motifs to target host domains that mediate DDIs exclusively in eukaryotes. H = host protein; B = generic bacterial protein; BE = bacterial effector protein. Domains are represented as blue (eukaryotic), red (bacterial), purple (eukaryotic-like and host-targeting), and black (convergently evolved and host-targeting) arcs. Short linear motifs are represented as rectangles.

Here, we constructed a domain-resolved network consisting of eukaryote-endogenous, bacteria-endogenous and host-bacteria protein-protein interactions (PPIs), based on which we studied the mechanism of host binding site mimicry by bacterial proteins, and systematically probed the proteomes of pathogenic bacteria for domains that mimic or target host domains engaging in domain-domain interactions (DDIs) that are specific to eukaryotes, as opposed to DDIs that are conserved between eukaryotes and bacteria. Our comprehensive and quantitative profiling of bacterial proteomes reveals statistically significant enrichment of domains and short linear motifs in bacterial effectors that interact with host domains engaged in eukaryote-specific DDIs, which allows host-bacteria PPIs to mimic host-endogenous PPIs on an interactome scale (Figure 7). We found that consistent with previous results for host-virus interactions, binding site sharing among host proteins largely results from gene duplication followed by divergent evolution, whereas binding site mimicry by bacterial proteins seems to largely result from convergent evolution (or extreme divergent evolution) of structural modules in bacteria that bear little resemblance to those in host. Our results indicate that: 1) effectors are six times as likely as non-effectors to contain host-like domains that mediate DDIs exclusively in eukaryotes (Figure 2); 2) the average domain in effectors is seven times as likely as that in non-effectors to co-occur with DDI partners in eukaryotes rather than in bacteria (Figure 3); and 3) effectors are nine times as likely as non-effectors to contain bacteria-exclusive domains that target host domains mediating DDIs exclusively in eukaryotes (Figure 4). Moreover, in the absence of host-like domains or among pathogen proteins without domain assignment, effectors harbor a higher variety and density of short linear motifs targeting host domains that mediate DDIs exclusively in eukaryotes.

While our dataset does contain more host-endogenous than bacteria-endogenous or host-bacteria PPIs and DDIs, this imbalance should not confound our results, as domain assignment and DDI templates are not taxonomy-specific, but rather are used to resolve all PPIs, regardless of the species involved. In fact, our estimation of domain’s relevance to eukaryote-specific DDIs anticipates and accounts for DDIs that are exclusive to host species, by giving more weight to domains engaging in such DDIs. In Tables 1,3 showing examples of effectors containing domains mediating DDIs either exclusively or primarily in eukaryotes, the fact that many domains can be traced to a few species is a technical consequence of proteins containing the same domains being merged into UniRef50 clusters, and only the species of the representative member of each cluster being retained. It is also a testament to extensive domain sharing among diverse pathogenic species. Taxonomic information may be useful when comparing effectors that are indistinguishable at the domain level but exhibit more variations at the residue level. Pooled analysis of proteins with identical domain compositions across different species can reveal general patterns in the host-bacteria PPI network that may not be obvious on a species-by-species or protein-by-protein basis. On the one hand, host domains targeted by multiple effector domains can reveal convergent evolution of common virulence mechanisms among different pathogenic species, which may prove useful in developing broad spectrum antibiotics. For instance, the human Ras domain (PF00071) is targeted by structurally distinct domains in *Legionella* (PF14860, PF18172, PF18641), *Pseudomonas* (PF03496), *Salmonella* (PF03545, PF05925, PF07487), *Shigella* (PF03278) and *Yersinia* (PF00069, PF09632) effectors. On the other hand, effector domains targeting multiple host domains and thus potentially perturbing multiple host pathways represent targets for multipronged therapeutic intervention. Of the 103 host-targeting bacterial proteins in our PPI dataset, 71 interact with a single host protein, while 32 interact with multiple host proteins. For instance, the *Pseudomonas* effector ExoS contains the ADP ribosyltransferase domain (PF03496), which it uses to target host proteins containing either a 14-3-3 domain (PF00244) or Ras small GTPase domain (PF00071). These host domains participate in a wide array of signaling pathways (Xiao et al., 1995; Stenmark and Olkkonen, 2001). While experimentally determined protein-protein interactions (PPIs) may be biased towards well-studied species, and domain-domain interaction templates may be biased towards well-studied protein structures, our survey of the proteomes of 84 pathogenic bacterial species is nonetheless more comprehensive than case studies of bacterial effectors in uncovering general molecular recognition principles underlying the host-bacteria PPI network. To increase coverage of the structurally-resolved host-bacteria PPI network, future efforts should focus on emerging pathogenic strains of bacteria, more systematic mapping of host-bacteria interactomes, as well as new molecular modelling methods to predict structures of proteins and protein-protein interactions which do not have homologs with known structure (Wu and Zhang, 2008).

To identify domains in bacteria that are most likely involved in mimicking host-endogenous protein-protein interactions (PPIs), we excluded eukaryotic-like domains which engage in either interchain or intrachain domain-domain interactions (DDIs) in bacteria. Previous studies suggest that intrachain DDIs often occur between adjacent domains within the same protein (Littler and Hubbard, 2005); however, PPIs are much less likely attributable to DDIs derived solely from intrachain interactions, compared to DDIs derived from interchain interactions (Itzhaki et al., 2006). Although distinguishing biologically relevant interfaces from artifactual crystal contacts is beyond the scope of this work, several interface classification algorithms have been developed to address this specific issue, based on various criteria such as contact size and evolutionary conservation of interface residues (Valdar and Thornton, 2001; Duarte et al., 2012), thermodynamic prediction of interface stability (Krissinel and Henrick, 2007), and interface conservation across multiple crystal forms of a protein (Xu et al., 2008). Here, we take a conservative approach and exclude all eukaryotic-like domains engaging in intraprotein DDIs in bacteria, because while conserved eukaryotic-like DDIs may not contribute to homologous PPIs in bacteria, they may function in maintaining protein stability or metabolic processes in bacteria (Tsoka and Ouzounis, 2000), rather than mediate host-bacteria PPIs. One example of a domain mediating PPIs in eukaryotes but serving a structural function in bacteria is the Fibronectin type III domain (PF00041), which in animals is involved in cell adhesion, migration and differentiation, and whose interaction with 44 domains leads to 1,156 interactions among 738 proteins in eukaryotes. While PF00041 does not mediate PPIs between bacterial proteins, it forms crystal contacts with the domain PF00704 within the *Bacillus thuringiensis* chitinase protein (PDB: 6BT9), and likely acts as a linker in the multi-domain chitinase (Juarez-Hernandez et al., 2019), rather than mediating host-pathogen PPIs.

In conclusion, our demonstration of binding site mimicry and its mechanisms at the domain level in the host-bacteria PPI network provides novel insight into the evolution of host-interacting domains in bacterial effectors. In particular, we showed that convergent evolution (or extreme divergent evolution) appears to be the more dominant mechanism behind binding site mimicry in host-bacteria interactions. To date, similar analysis has only been done for viral proteins. In addition, our estimation of domain’s relevance to eukaryote-specific DDIs provides quantitative, interaction-based criteria for identifying novel effectors, based on: 1) domains that exclusively or primarily mediate DDIs in eukaryotes; and 2) variety and density of short linear motifs targeting host domains that exclusively mediate DDIs in eukaryotes. Although predicting new effector-host interactions is beyond the scope of this paper, our study presents a first step toward resolving PPI interfaces in effector-host interactions: once the domains involved in effector-host PPIs are identified by our method, interface residues inside such domains can be predicted using machine learning methods (Meyer et al., 2018). By mapping the interface residues involved in host-effector PPIs, it may be possible to develop antibiotics that precisely inhibit host-pathogen PPIs, with minimal disruption to host-endogenous PPIs (Voter and Keck, 2018). Given the scarcity of host-bacteria PPI data and the rapidly increasing number of completely sequenced pathogen genomes, our framework for assessing the functional impact of structural modules within pathogen proteins, without needing direct experimental evidence of their interaction with host proteins, may help accelerate the discovery and mechanistic study of novel virulence factors, as well as the development of selective inhibitors of pathogen-subverted host signaling pathways.

## Materials and Methods

### Domain-Resolved Eukaryote-Bacteria Protein-Protein Interaction Network

Eukaryote-endogenous, bacteria-endogenous, and host-bacteria protein-protein interaction (PPI) data were obtained from IntAct and HPIDB 3.0 (Orchard et al., 2014; Ammari et al., 2016). Domain-domain interaction (DDI) templates were obtained from 3did and Pfam (Mosca et al., 2014; El-Gebali et al., 2019). IntAct is one of the largest and most cited databases of literature-curated, high quality molecular interactions in multiple organisms (615,015 unique binary protein-protein interactions in 1,572 organisms). 3did is a comprehensive, regularly maintained resource for domain-domain and domain-motif interaction templates derived from PDB structures (14,278 domain-domain and 920 domain-motif interaction templates).

To resolve protein-protein interactions into domain-domain interactions, we first predicted the occurrence of domains in proteins with InterProScan, using Pfam’s gathering threshold (Jones et al., 2014). We then considered all 14,278 unique types of DDI templates involving 8,048 interacting Pfam domains in the 3did database, which corresponds to ∼1.77 DDIs per domain. The resulting integrated eukaryote-bacteria DDI network consists of 5,950 unique types of DDIs involving 3,558 interacting domains, which corresponds to ∼1.67 DDIs per domain. In other words, the DDI-to-domain ratio is roughly preserved in the construction of domain-resolved eukaryote-bacteria interactome. Possible reasons for the absence of certain domains and DDIs in our interactome are: 1) they only occur in organisms not considered in our study, such as archaea and viruses; and 2) the DDI only occurs between domains within the same protein, rather than mediating PPIs between different proteins. When a PPI can be attributed to several possible DDIs, priority was given to interchain DDIs (derived from PDB structures consisting of at least two distinct protein entities), followed by intrachain DDIs.

### Selection Criteria for Effector and Non-Effector Proteins

We included proteins encoded by pathogenic bacterial species catalogued in PHI-base (Urban et al., 2020). PHI-base contains expert curated, regularly updated data on pathogen genes with experimentally verified impact on host-pathogen interactions (216 host species, 274 pathogenic species, 7,681 pathogen genes). Effector protein IDs were retrieved from the PHI-base annotation file “phi-base_current.csv”, by searching for genes whose “Gene Function” or “Mutant Phenotype” column contains the keyword “effector”. In addition, effector protein IDs were also retrieved from UniProt (UniProt, 2019) using two sets of keywords. **By gene name:** taxonomy:“Bacteria [2]” name:effector (name:“type 1” OR name:“type 2” OR name:“type 3” OR name:“type 4” OR name:“type 5” OR name:“type 6” OR name:“type 7” OR name:“type 8” OR name:“type 9” OR name:t*ss OR name:“secretion system”) **By cellular location:** taxonomy:“Bacteria [2]” (annotation(type:function effector) OR locations: (note:“type 1”) OR locations(note:“type 2”) OR locations: (note:“type 3”) OR locations: (note:“type 4”) OR locations: (note:“type 5”) OR locations(note:“type 6”) OR locations: (note:“type 7”) OR locations: (note:“type 8”) OR locations: (note:“type 9”) OR locations: (note:t*ss) OR locations: (note:“secretion system”)) (locations: (location:“Secreted [SL-0243]”) OR locations: (location:“Host [SL-0431]”)) Non-effectors consist of cytoplasmic, membrane as well as other secreted proteins encoded by the same pathogen species considered for effector proteins. **Cytoplasmic:** taxonomy:“Bacteria [2]” locations: (location:“Cytoplasm [SL-0086]”) **Membrane:** taxonomy:“Bacteria [2]” (locations: (location:“Cell envelope [SL-0036]”) OR locations: (location:“Membrane [SL-0162]”)) **Secreted:** taxonomy:“Bacteria [2]” (locations: (location:“Secreted [SL-0243]”) OR locations: (location:“Host [SL-0431]”))

### Merging Bacterial Proteins With Identical Domain Compositions

Taxonomy and Pfam domain annotations of proteins were obtained from UniProt and InterPro (Mitchell et al., 2019). For each domain, we counted the number of eukaryotic and bacterial species encoding at least one protein containing that domain. To minimize the impact of spurious domains, such as arising from contaminated genomes or misannotated proteins, we required that each domain be found in at least three eukaryotic or bacterial proteomes–at least one of which must be a reference proteome or belong to a pan proteome. Bacterial proteins with identical domain compositions were merged into a single entry, as they are indistinguishable from one another at the domain resolution. To further reduce redundancy among highly related protein sequences (*e.g.* orthologs or fragments of the same protein) while also maintaining sufficient resolution, sequences belonging to the same UniRef50 cluster were ranked based on whether they are: 1) representative for the cluster, as assigned by UniRef50; 2) manually reviewed; 3) assigned high annotation score by UniProt; 4) from UniProt reference proteomes; and 5) longest. Only the top-ranking sequence was retained for each UniRef50 cluster. For domain compositions that are common to effectors and non-effectors, we assessed their relative frequency in effectors *vs.* non-effectors. Domain compositions that are significantly enriched (*q*-value < 0.1) in effectors were assigned to effectors, and domain compositions that are significantly depleted (*q*-value < 0.1) in effectors were assigned to non-effectors. Our final dataset thus contains 238 effectors and 3,921 non-effectors with unique domain signatures.

### Statistical Tests Performed

Fisher’s exact test was used for analyses based on odds ratios, and Wilcoxon test was used for analyses based on difference in means. To control the false discovery rate in multiple hypothesis testing, we calculated the positive false discovery rate, or q-values (Storey, 2003). All statistical analyses were conducted in R (Team, 2018).

## Data Availability

Publicly available datasets were analyzed in this study. This data can be found here: IntAct (ftp://ftp.ebi.ac.uk/pub/databases/intact/current/psimitab/intact.zip) HPIDB (http://hpidb.igbb.msstate.edu/downloads/hpidb2.mitab.zip) InterPro (ftp://ftp.ebi.ac.uk/pub/databases/interpro/uniparc_match.tar.gz) Eukaryotic Linear Motif (http://elm.eu.org/downloads.html).
